# Towards Semi-Automatic Artifact Rejection for the Improvement of Alzheimer’s Disease Screening from EEG Signals

**DOI:** 10.3390/s150817963

**Published:** 2015-07-23

**Authors:** Jordi Solé-Casals, François-Benoît Vialatte

**Affiliations:** 1 Data and Signal Processing Research Group, University of Vic–Central University of Catalonia, Vic, 08500 Barcelona, Spain; E-Mail: jordi.sole@uvic.cat; 2 BCI Team, Brain Plasticity Laboratory, UMR 8249, CNRS, Paris 75005, France; 3 ESPCI ParisTech, PSL Research University, Paris 75005, France

**Keywords:** EEG, artifacts, blind source separation, Alzheimer’s disease, screening

## Abstract

A large number of studies have analyzed measurable changes that Alzheimer’s disease causes on electroencephalography (EEG). Despite being easily reproducible, those markers have limited sensitivity, which reduces the interest of EEG as a screening tool for this pathology. This is for a large part due to the poor signal-to-noise ratio of EEG signals: EEG recordings are indeed usually corrupted by spurious extra-cerebral artifacts. These artifacts are responsible for a consequent degradation of the signal quality. We investigate the possibility to automatically clean a database of EEG recordings taken from patients suffering from Alzheimer’s disease and healthy age-matched controls. We present here an investigation of commonly used markers of EEG artifacts: kurtosis, sample entropy, zero-crossing rate and fractal dimension. We investigate the reliability of the markers, by comparison with human labeling of sources. Our results show significant differences with the sample entropy marker. We present a strategy for semi-automatic cleaning based on blind source separation, which may improve the specificity of Alzheimer screening using EEG signals.

## 1. Introduction

Alzheimer’s disease (AD) is the most common type of dementia among the elderly; its socioeconomic cost to society is sizeable and expected to increase. It is characterized by progressive and irreversible cognitive deterioration with memory loss, impairments in judgment and language, and other cognitive deficits and behavioral symptoms that finally become severe enough to limit the ability of an individual to carry out the professional, social or family activities of daily life. As the disease progresses, patients develop increasingly severe disabilities, becoming in the end completely dependent on others. An early and accurate diagnosis of AD would be of much help to patients and their families, both in facilitating planning for the future and in beginning treatment of the symptoms of the disease early. 

A diagnosis of AD requires, on the one hand, the confirmation of the presence of a progressive dementia syndrome and, on the other hand, the exclusion of other potential causes of dementia as demonstrated by the patient’s clinical history. An unambiguous diagnosis of AD is considered to require that a post-mortem analysis demonstrate the typical AD pathological changes in brain tissue [1,2,3]. The clinical hallmark of the earliest manifestations of AD is episodic memory impairment. At the time of clinical presentation, other cognitive deficits are usually already present in the patient’s language, executive functions, orientation, perceptual abilities and constructional skills. Associated behavioral and psychological symptoms include apathy, irritability, depression, anxiety, delusions, hallucinations, inhibition decrease, aggression, aberrant motor behavior, as well as changes in eating or sleeping patterns [4,5]. While the presence of these symptoms is indicative of AD, reaching a reliable diagnosis in some cases requires expensive and invasive diagnostic tests such as computer tomography (CT), magnetic resonance imaging (MRI) and/or lumbar puncture. 

In order to develop a system for an early diagnosis of AD, the potential of a recording technique known as electroencephalography (EEG) has been investigated. EEG consists in recording brain-related electrical potentials using different electrodes attached to the scalp [6]. EEG activity is commonly divided into specific frequency bands: 0.1 Hz–4 Hz (δ), 4 Hz–8 Hz (θ), 8 Hz–13 Hz (α), 13 Hz–30 Hz (β) and 30 Hz–100 Hz (γ)[6]. A large number of studies have analyzed measurable changes that AD causes on EEG. A review of these studies can be found in [7,8,9]. Three major perturbations have been reported in EEG: (i) power increase of δ and θ rhythms and power decrease of posterior α and/or β rhythms in AD patients (also known as EEG slowing); (ii) EEG activity of AD patients seems to be more regular than the EEG recording of healthy subjects (which correspond to reduced complexity of the EEG signals for AD patients); and (iii) frequency-dependent abnormalities in EEG synchrony [7,8,10]. Despite being easily reproducible, those markers have limited sensitivity, which is for a large part due to the poor signal-to-noise ratio of EEG signals.

EEG recordings are indeed usually corrupted by spurious extra-cerebral artifacts, which should be rejected or cleaned up by the practitioner. These artifacts are responsible for a consequent degradation of the signal quality. In previous works we presented several methodologies to improve the quality of EEG data in patients with AD using blind source separation (BSS) [11,12]. BSS appears to be more suitable for artifact rejection than adaptive filtering and regression [7]. However, BSS is not fully automatic: one needs to visually inspect the components extracted by BSS and decide which components to remove; this time consuming process is not suitable for routine clinical EEG [13]. Furthermore, visual inspection is subjective [14], and the reliability of BSS is therefore limited.

Since manual screening of human EEGs is inherently error prone and might induce experimental bias, automatic artifact detection is an issue of importance. It is most certainly one of the keys to achieve reliable diagnostics, and obtain useful results for clinical purposes [7]. Automatic artifact detection would consequently be the best guarantee for objective and clean results. Unfortunately, automatic detection is fairly difficult to perform, due to the lack of reliable markers of EEG artifacts. The evaluation of a given set of markers could be performed using either simulated EEG recordings, or real EEG recordings. In the first case, the content of the signals is well defined; however, one cannot guarantee that the signals investigated are comparable with real signals. In the second case, one cannot guarantee that the artifacts are well identified, knowing that EEG studies usually reach fairly poor inter-expert agreements. In the present investigation, we chose the second option. We present here an investigation of commonly used markers of EEG artifacts. We investigate the possibility to automatically clean a database of EEG recordings taken from AD patients and healthy age-matched controls. The data is first decomposed using BSS, and afterwards artifacted sources are rejected. In order to reduce risks coming from the poor human inter-expert agreements, data is not screened by only one expert, but by three independent experts to locate artifacts within the sources. Due to the importance of not rejecting EEG data, the approach is conservative, in the sense that as a rule data is not eliminated if it’s not clearly identified as an artifact. We afterwards investigate the reliability of the markers, by comparing the markers with the resulting human labeling of sources (sources identified as artifacts *versus* sources identified as clean EEG). 

## 2. Results

We first compared the number of rejected sources from the control and Alzheimer groups (Table 1) with a Wilcoxon ranksum test, taking into account a Bonferroni-corrected significant threshold of $p_{max}=1.67\times 10^{-2}$ (equivalent to *p* = 0.05 without correction). None of the experts showed a significant difference of treatment between the groups (the smallest p-value was 0.06).

**Table 1:** Statistical comparison of the number of sources rejected in the group of 24 control subjects *versus* the group of 17 patients suffering from Alzheimer’s disease (two-sided Wilcoxon ranksum test) for each expert considered independently, and all experts aggregated. Rows indicate the number of rejected sources for AD patients (average and standard deviation), the number of rejected sources for control subjects, p-values (*p* < 0.05 indicates a significant difference) and the Wilcoxon z-score statistic.

	Expert #1	Expert #2	Expert #3	All
NAD	5.0±1.5	3.9±1.2	5.4±1.2	4.8±1.4
NCTR	5.9±1.1	4.1±1.5	5.1±1.4	5.0±1.5
p	0.06	0.78	0.52	0.32
z	−1.90	−0.27	0.64	−0.99

We afterwards compared the power of the four features (Table 2) with a Wilcoxon ranksum test, taking into account a Bonferroni-corrected significant threshold of $p_{max}=1.25\times 10^{-2}$ (equivalent to *p* = 0.05 without correction). Whether on patients suffering from Alzheimer’s disease, control subjects or when aggregating all subjects, sample entropy always appears as the most powerful feature, with the strongest Z-score and the lowest p-value (overall, below $1.75\times 10^{-4}$). All the features are significant, except for K, which is non-significant for the control group. Group effects comparing rejected sources between Alzheimer and Control groups and the clean sources between Alzheimer and Control groups were systematically improved by artifact rejection. In particular the cleaned sources had very significant differences with the zero-crossing and kurtosis measure (*p* = $4.04\times 10^{-30}$ and *p* = $8.80\times 10^{-29}$ respectively).

**Table 2:** Statistical comparison of the features for artifacts sources *versus* clean sources (two-sided Wilcoxon ranksum test) for the group of 17 patients suffering from Alzheimer’s disease, for the group of 24 control subjects, for all 41 subjects aggregated, and group effects comparing rejected sources between Alzheimer and Control groups and the clean sources between Alzheimer and Control groups. All measures from the three experts were aggregated for this test. Rows indicate p-values and the Wilcoxon z-score statistic. Gray background indicates the most powerful feature (sample entropy).

**Alzheimer**		**SEnt**	**FD**	**K**	**Z**
	**P**	$1.75\times 10^{-4}$	$1.80\times 10^{-3}$	$2.10\times 10^{-3}$	$2.00\times 10^{-3}$
	**Z**	3.75	3.12	−3.07	3.08
**Control**	**P**	$7.48\times 10^{-6}$	$3.00\times 10^{-3}$	$7.23\times 10^{-2}$	$2.37\times 10^{-5}$
	**Z**	4.48	2.96	−1.80	4.23
**All**	**p**	$1.39\times 10^{-9}$	$1.60\times 10^{-5}$	$2.30\times 10^{-3}$	$1.62\times 10^{-7}$
	**z**	6.06	4.31	−3.05	5.24
**Rejected sources**	**p**	$1.50\times 10^{-3}$	$3.60\times 10^{-3}$	$5.24\times 10^{-4}$	$2.99\times 10^{-9}$
	**z**	−3.18	−2.91	−3.47	−6.30
**Cleaned sources**	**p**	$5.99\times 10^{-9}$	$2.44\times 10^{-6}$	$8.80\times 10^{-29}$	$4.04\times 10^{-30}$
	**z**	−5.82	−4.71	−11.13	−11.40

We evaluated the capabilities of these methods to automatically detect EEG artefacts using a classification approach (Table 3). A multilayer perceptron was trained on half of the database, and tested on the spared samples. The purpose was to classify rejected sources from non-artifacted sources. As we can see, the classifier can generalize this classification, despite the performances being moderate. 

Knowing that experts do not always agree on source selection, we controlled for the possible effects of the experts. To that extent, we compared again the power of the four features (Table 4) with a Wilcoxon ranksum test, and the group effect of differences between experts with a Kruskall-Wallis test, taking into account a Bonferroni-corrected significant threshold of $p_{max}=1.25\times 10^{-2}$. Whatever the expert, sample entropy always appears as the most powerful feature, with the strongest Z-score and the lowest p-value (overall, p is always below $1.10\times 10^{-3}$). All the features are significant, except for FD and K, which are non-significant for experts #1 and #3 and for all experts respectively. There was no group effect of difference between experts.

**Table 3:** Automatic detection of artifacts sources *versus* clean sources (multilayer perceptron with 2-fold cross-validation) for each expert considered independently and aggregated together. Classification was performed for sources from the group of 17 patients suffering from Alzheimer’s disease, and for sources from the group of 24 control subjects. Rows indicate the classification rate average and standard deviation on 1000 classification attempts.

	Control Subjects	Alzheimer Patients
**Expert #1**	62.8%±5.0%	61.6%±6.4%
**Expert #2**	65.5%±6.6%	63.9%±7.5%
**Expert #3**	59.0%±5. 5%	65.1%±4.0%
**All**	64.8%±3.1%	65.7%±3.6%

**Table 4:** Statistical comparison of the features for artifacts sources *versus* clean sources (two-sided Wilcoxon ranksum test) for each expert considered independently, and group effects comparing rejected sources between experts and the clean sources between experts (Kruskal-Wallis test). All subjects were aggregated for this test. Rows indicate p-values and the Wilcoxon z-score or Kruskal-Wallis Chi² statistics. Gray background indicates the most powerful feature (sample entropy).

**Expert #1**		**SEnt**	**FD**	**K**	**Z**
	**P**	$1.10\times 10^{-3}$	$2.83\times 10^{-2}$	$1.73\times 10^{-2}$	$7.40\times 10^{-3}$
	**Z**	3.25	2.19	−2.38	2.68
**Expert #2**	**P**	$7.51\times 10^{-5}$	$4.04\times 10^{-4}$	$9.11\times 10^{-2}$	$1.10\times 10^{-3}$
	**z**	3.96	3.54	−1.69	3.26
**Expert #3**	**p**	$6.44\times 10^{-4}$	$5.82\times 10^{-2}$	$2.25\times 10^{-1}$	$1.00\times 10^{-3}$
	**z**	3.41	1.89	−1.21	3.28
**Rejected sources**	**p**	0.18	0.11	0.82	0338
	$\chi^{2}$	1.83	2.52	$4.93\times 10^{-2}$	0.93
**Cleaned sources**	**p**	0.32	0.99	0.52	0.43
	$\chi^{2}$	0.97	$5.82\times 10^{-6}$	0.41	0.62

**Figure 1:**
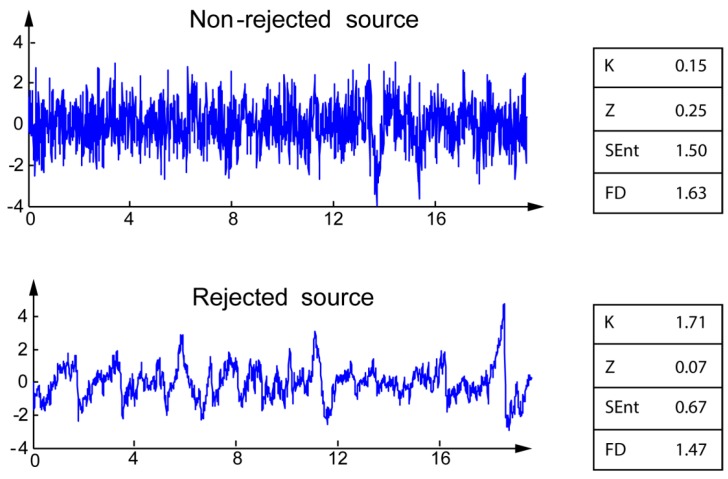
Illustration of the four markers for non-rejected (**top**) and rejected (**bottom**) sources.

Most of the artifacts identified by the experts are typical of EEG signals: eye blinks, eye movements, EKG, and other unidentified (artifacts with unclear pattern). The differences between the four markers for rejected and non-rejected sources are illustrated on typical examples on Figure 1. For the rejected sources, Kurtosis K is higher, whereas Zero-crossing rate Z, Sample entropy, and fractal dimension are lower than those of non-rejected sources.

When comparing the control and patient groups before and after the procedure, the Leave-One-Out root mean square error (RMSE) of validation dropped from 0.32 to 0.28 (training RMSE dropped from 0.30 to 0.26). Classification of EEG relative powers after the artifact cleaning procedure was more efficient than before cleaning.

## 3. Discussion

We investigated markers that could be used to automatized the three source removal criteria described in Section 4.3, in order to avoid any manual screening and reduce human errors. The artifact cleaning procedure led to an improved detection of AD, with a systematic improvement of the differences before and after cleaning (see Table 2) and a classification error dropping from 32% to 28%. The first and second criteria are easy to automatize because are directly related to the EEG signals on the electrodes (first criterion) and estimating mixing matrix (second criterion). The first rule, “Source of abnormally high amplitude (≥100 µV)”, is easy to implement by thresholding the backpropagated sources and eliminating those with peaks over 100 µV. The second rule is related to the detection of isolated sources on the scalp: “Abnormal scalp distribution of the reconstructed channels (only a few electrodes contribute to the source, with an isolated topography)”. In order to simplify the detection of isolated IC on the scalp we can use the information given by the inverse of the de-mixing matrix obtained after the decomposition by EWASOBI algorithm. By calculating the inverse of this matrix we obtain an estimated mixing matrix that can allow us to find the back projection of the source n onto the original data (electrodes domain). Then, we can calculate the energy that each electrode contributes to this n source and label as an artifact if the percentage of the energy of one (or very few) electrode(s) is higher than a pre-fixed threshold (50% of the total energy, for example). 

The third rule, “Abnormal wave shape (drifts, eye blinks, sharp waves, *etc.*)”, is a real challenge, and was the main object of our investigation. Our working hypothesis was that this rule could be implemented based on the statistical properties of the time series. However, note that we do not show the statistic for the third rule only, but for the global combination of the three rules: indeed, almost all the rejected sources are selected by the experts with the combined application of the three rules. For instance, a source with sufficiently high amplitude and a sufficiently abnormal shape was rejected by most experts. However, a source with slightly high amplitude and normal shape may not be rejected by all three experts (it is on those sources that the experts will not always reach a consensus). We investigated in this manuscript four potential statistical markers in order to characterize the time series, which could provide some information about potentially abnormal shapes in the EEG sources. Our observations are congruent with the existing literature. Kurtosis K is higher for the artifacted source: their distributions are farther from Gaussianity than non-rejected sources [15]. Despite this effect being well known, kurtosis was the poorest marker in our study, confirming previous results of Delorme *et al.* [15]. Sample entropy is lower for rejected sources, owing to the increased predictability of the repetitive artefact patterns. This result is congruent with the literature: the expected SEnt of artifacts is lower, because their patterns are more regular and predictable in comparison with neural activity [16]. Similarly, FD is lower for artifacted sources, in accordance with previous publications: clean EEG traces are typically characterized by a flatter and more spread spectrum, with higher FD [17,18], especially for ocular artifacts [19]. Zero-crossing rate Z is lower for the artifacted sources. Unfortunately, despite several studies reporting the use of zero-crossings for the evaluation of artifacts in EEG signals, to the best of our knowledge, none of them reported if this measure increases or decreases. We can nevertheless conjecture that our observation is valid, and can be explained by the presence of low-frequency perturbations forming blocks in the presence EEG artifacts (which is compatible with the effects observed with SEnt and FD).

Automatic classification of the sources leads to an accuracy of ~65% on the validation set depending on the expert involved in source selection. Despite this result is not sufficient to guarantee an efficient automatic rejection, it clearly demonstrates the potential of these measures for semi-automatic rejection. Indeed, by tweaking the classification threshold, one can obtain a higher sensitivity to the detriment of a lower specificity (sensitivity of 80.0% for a specificity of 26.0% using a linear classifier). In other words, the classifier can automatically detect a subset of suspicious sources, and thereby alleviate the task of the expert who will only need to remove the non-artifacted sources from this selection. Taking into account the fact that the expert labeling is error prone, these results are encouraging.

## 4. Material and Methods

### 4.1. EEG Data—Patients with MildAD

These data were obtained using a strict protocol from Derriford Hospital, Plymouth, UK and had been collected using normal hospital practices [20]. EEGs were recorded during a resting period with various states: awake, drowsy, alert and resting states with eyes closed and open. All recording sessions and experiments proceeded after obtaining the informed consent of the subjects or the caregivers and were approved by local institutional ethics committees. EEG dataset is composed of 24 healthy control subjects (age: 69.4 + 11.5 years old; 10 males) and 17 patients with mild AD (age: 77.6 + 10.0 years old; 9 males). The EEG time series were recorded using 19 electrodes disposed according to Maudsley system, similar to the 10–20 international system, at a sampling frequency of 128 Hz. EEGs were band-pass filtered with digital 2nd order Butterworth filter (forward and reverse filtering) between 0.5 and 30 Hz (a sampling rate of 128 Hz means that frequencies above 25 Hz cannot be reliably studied [21]).

### 4.2. BSS Algorithm

Blind Source Separation (BSS) consists in recovering a set of unknown sources s from their observed mixture **x**. The linear and instantaneous models of BSS can be formulated as:

(1) x = As

where s represents a data matrix having as rows the unknown sources, and A is the mixing matrix. According to the currently prevailing view of EEG signal processing, a signal can be modeled as a linear mixture of a finite number of brain sources, with additive noise (see e.g., [13,22,23]). Therefore, blind source separation techniques can be used advantageously for decomposing raw EEG data to brain signal subspace and noise subspace. If sources are supposed to be independent, then BSS can be called ICA.

The Second-Order Blind Identification (SOBI) algorithm is a well-known blind source separation (BSS) method for source signals with temporal structures and distinct spectra (AR processes). It already proved to be useful in many biomedical applications. A weight-adjusted version of SOBI was suggested in [24]. SOBI jointly (approximately) diagonalizes time-delayed covariance matrices for many time delays. However, SOBI algorithm does not specify how many and which time delays to choose. An efficient weight adjusted variant of SOBI called IWASOBI [25,26] was developed to solve this problem. The original weight adjusted SOBI used a standard AJD (Approximate Joint Diagonalization) algorithm. IWASOBI uses instead an AJD based on family of WEDGE1 algorithms [25]. For IWASOBI the number of jointly diagonalized covariance matrices can be relatively low in comparison to the standard SOBI while performance can be considerably higher. This algorithm allows reliable separation of 100+ sources with temporal structure (autoregressive sources) in order of seconds. In our experiments we used the IWASOBI algorithm implemented in ICALAB ver.3 [27].

### 4.3. Artifact Removal Procedure

**Figure 2:**
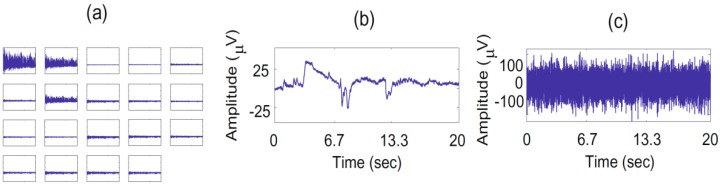
Examples of artifacts: (**a**) abnormal scalp distribution of the reconstructed channels; (**b**) abnormal wave shape; (**c**) source of abnormally high amplitude.

The present work extends our preliminary results presented in [12]. Each recording under analysis is decomposed using IWASOBI. Sources are ordered using a kurtosis measure, and then some of them corresponding to artifacts (eye movements, EMG corruption, EKG, *etc.*) are removed after visual inspection by three independent experts, according to the following criteria (illustrated on Figure 2):

- Source of abnormally high amplitude (≥100 µV in the back projected EEG signal).
- Abnormal scalp distribution of the reconstructed channels (only a small subset of electrodes contribute to the source, with an isolated topography).
- Abnormal wave shape (drifts, eye blinks, sharp waves, *etc.*).

The maximum of source that are removed is limited to one third of the total number, in order to prevent denaturing EEG signals. On average, 4.8 ± 1.4 sources were rejected for AD patients, and 5.0 ± 1.5 for control subjects (the difference is not significant, see also Table 1).

### 4.4. Statistical Markers of the EEG Shape

We investigated the set of statistical markers listed below (Table 5), which are good candidates for artifact detection. We computed the values of all independent components (artifact-labelled and clean-labelled components) for all these markers, using Matlab^®^ R2013a. 

**Table 5:** Names and acronyms of the considered statistical markers.

Name	Acronym
Kurtosis	K
Zero-crossing	Z
Sample entropy	SEnt
Fractal dimension	FD

For a time series X with n samples $\left[x_{1},x_{2},\ldots ,x_{n}\right]$, we can define the Kurtosis *K* as follows:

(2) $$\text{K}=\frac{{\text{μ}}_{4}}{{\text{μ}}_{3}}-3=\;\frac{\frac{1}{\text{n}}\sum_{\text{i}=1}^{\text{n}}{\left({\text{x}}_{\text{i}}-\bar{\text{x}}\right)}^{4}}{{\left\{\frac{1}{\text{n}}\sum_{\text{i}=1}^{\text{n}}{\left({\text{x}}_{\text{i}}-\bar{\text{x}}\right)}^{2}\right\}}^{2}}-3$$

where $\bar{x}$ is the mean of X, and ${\text{μ}}_{4}$ and ${\text{μ}}_{3}$ are respectively the fourth and third central moments. The Kurtosis is the degree of peakedness of a distribution, and is a well-known indicator of the potential presence of artifacts EEG signals [15,28,29,30]. For normally distributed data, the kurtosis is zero. If the distribution function of the data has a flatter top than the normal distribution, then the kurtosis is negative. The kurtosis is positive if the distribution function has a high peak compared to the normal distribution.

The zero-crossing rate ZC measures the number of times the signal crosses the abscissa:

(3) $$\text{ZC}=\frac{1}{\text{n}-1}\overset{\text{n}-1}{\underset{\text{i}=1}{\sum }}I\left({\text{x}}_{\text{i}}{\text{x}}_{\text{i}+1}<0\right)$$

where the indicator function I(A) is 1 if its argument A is true and 0 otherwise. This rate may be used as a marker to indicate the presence of artifacts in EEG [28,29,30].

The sample entropy is defined for a time series of *n* points. We first define the n−m+1 vectors $x_{m}\left(i\right)=\left\{u\left(i+k\right):o\leq k\leq m-1\right\},$ as the vectors of *m* data points from u(i) to u(i+m−1). The distance between two such vectors is defined to be $d\left[x_{m}\left(i\right),x_{m}\left(j\right)\right]=\underset{k}{\max}\left\{\left|u\left(i+k\right)-u\left(j+k\right)\right|:o\leq k\leq m-1\right\}$, the maximum difference of their corresponding scalar components. The sample entropy statistic **SEnt** is defined as:

(4) $$\text{SEnt}\left(\text{m},\text{r}\right)=\underset{\text{n}\to \infty }{\lim}\left\{-\text{ln}\left({\text{A}}^{\text{m}}\left(\text{r}\right)/{\text{B}}^{\text{m}}\left(\text{r}\right)\right)\right\}=-\text{ln}\left(\text{A}/\text{B}\right)$$

with

(5) $$\text{A}=\left[\left(\text{n}-\text{m}-1\right)\left(\text{n}-\text{m}\right)/2\right]{\text{A}}^{\text{m}}\left(\text{r}\right)$$

and

(6) $$\text{B}=\left[\left(\text{n}-\text{m}-1\right)\left(\text{n}-\text{m}\right)/2\right]{\text{B}}^{\text{m}}\left(\text{r}\right)$$

$B^{m}\left(r\right)$ is the probability that two sequences match for m points:

(7) $${\text{B}}^{\text{m}}\left(\text{r}\right)={\left(\text{n}-\text{m}\right)}^{-1}\overset{\text{n}-\text{m}}{\underset{\text{i}=1}{\sum }}{\text{B}}_{\text{i}}^{\text{m}}\left(\text{r}\right)$$

where $B_{i}^{m}\left(r\right)$ is ${\left(n-m-1\right)}^{-1}$ times the number of vectors $x_{m}\left(j\right)$ within r of $x_{m}\left(i\right)$. Similarly, $A^{m}\left(r\right)$ is the probability that two sequences match for m+1 points:

(8) $${\text{A}}^{\text{m}}\left(\text{r}\right)={\left(\text{n}-\text{m}\right)}^{-1}\overset{\text{n}-\text{m}}{\underset{\text{i}=1}{\sum }}{\text{A}}_{\text{i}}^{\text{m}}\left(\text{r}\right)$$

where $A_{i}^{m}\left(r\right)$ is ${\left(n-m-1\right)}^{-1}$ times the number of vectors $x_{m+1}\left(j\right)$ within r of $x_{m+1}\left(i\right)$. The scalar r is the tolerance for accepting matches. In the present investigation, we used the parameters recommended in [31], with m=2 and r=0.2 (standard deviation of the sources is normalized to 1). SEnt is a robust quantifier of complexity in EEG signals [32], and can be used as a marker for the presence of artifacts in EEG recordings [16].

Sevcik showed that the fractal dimension FD of a curve can be approximated from its Hausdorff dimension h [33]:

(9) $$\text{Dh}=\underset{\text{ε}\to 0}{\lim}\frac{-\log\left(\text{N}\left(\text{ε}\right)\right)}{\log\left(\text{ε}\right)}$$

where N(ε) is the number of open balls of radius ε needed to cover the set. In a metric space, given any point p, an open ball of radius ε is a set of all points q for which dist(p,q)<ε. A curve of length L may be divided into $N\left(\text{ε}\right)=\frac{L}{2\text{ε}}$ segments of length 2ε and may be covered by N(ε) balls of radius ε. Consequently the expression becomes:

(10) $$\text{Dh}=\underset{\text{ε}\to 0}{\lim}\left[1-\frac{\log\left(\text{L}\right)-\log\left(2\right)}{\log\left(\text{ε}\right)}\right]$$

Sevcik proposes a double linear transformation of the curve into another normalized metric space, making all axes equal since the topology of a metric space does not change under linear transformation. After this normalization, and taking $\text{ε}=\frac{1}{2n\prime }$ and $n^{\prime }=n-1$, the above equation becomes:

(11) $$\text{FD}=\underset{\text{n′}\to +\infty }{\lim}\left[1+\frac{\log\left(\text{L}\right)-\log\left(2\right)}{\log\left(2\text{n′}\right)}\right]$$

The measure of Sevcik is approximately equal to the fractal dimension and the approximation improves as n′→+∞. The analysis of fractal dimension can be used for artifact detection in EEG signals [17,18,19].

### 4.5. Classifications

In order to control for the predictive power of the statistical markers, we designed a multilayer perceptron [34]. The model classified the data samples using the statistical markers into rejected or non-rejected sources. We used a cross-validation procedure: the database was divided into two halves, and the network was trained on the first half (training set) and tested on the other half (validation set). In order to avoid local minima, this procedure was repeated with 1000 re-initializations, and the average validation error was estimated.

In order to control for the effect of the artifact rejection procedure, we aggregated the Fourier power of each patient and each control subject into five regions (frontal, temporal left and right, central and occipital). Linear discriminant analysis was applied before and after the artifact rejection procedure. We estimated a Leave-One-Out error [23]: iteratively one subject or patient was removed from the database. Afterwards, the classifier was trained on the remaining data. Finally, the classifier was tested on the removed sample (used as a validation sample). The Leave-One-Out score was the root mean square error for all the subjects and patients in the database.

## 5. Conclusions

Our results confirm that SEnt was the best of the four investigated markers. As stated in the introduction, the presence of artifacts in EEG is one of the main reasons of its limited specificity for the diagnostic of Alzheimer’s disease. SEnt was previously shown to decrease significantly for patients suffering from Alzheimer, and could be used as a marker of the pathology [35]. Obviously, the presence of artifacts (which, as we have shown, decreases significantly SEnt measurements) blurs out the differences between recordings of control and patient groups. Removing artifacts could therefore prevent this blurring effect, and improve the specificity of EEG-based diagnostics. 
